# Chlorido{2-[(dimethyl­amino)­methyl]phenyl-κ^2^ *C* ^1^,*N*}(1-methyl-1*H*-imidazole-κ*N* ^3^)palladium(II)

**DOI:** 10.1107/S1600536810047367

**Published:** 2010-11-24

**Authors:** Jason W. Clements, Milorad Stojanovic, Norris W. Hoffman, Richard E. Sykora

**Affiliations:** a Department of Chemistry, University of South Alabama, Mobile, AL 36688-0002, USA

## Abstract

In the title compound, [Pd(C_9_H_12_N)Cl(C_4_H_6_N_2_)], which was synthesized from the reaction of 1-methyl­imidazole with dimeric dichloridobis[2-(dimethyl­amino)­benz­yl]palla­dium(II), the ring-deprotonated *N*,*N*-dimethyl­benzyl­amine ligand acts in a *C*,*N*-bidentate fashion. The dihedral angle between the ring of the 1-methyl­imidazole ligand and the palladacycle plane is 57.88 (16)°. The two N atoms from the *N*,*N*-dimethyl­benzyl­amine and 1-methyl­imidazole ligands are *trans* coordinated to the Pd^II^ atom.

## Related literature

For an overview of the application of palladacycles in organic synthesis, see: DuPont & Flores (2009 ▶); Bedford *et al.* (2003 ▶); Fors & Buchwald (2010 ▶). For detoxification of phospho­rothio­nate pesticides, see: Lu *et al.* (2010 ▶). For studies converting the dimeric precursor (Cope & Friedrich, 1968 ▶) of the title compound into monomeric square-planar palladacycles, see: Mentes & Büyükgüngör (2004 ▶); Mentes *et al.* (2004 ▶); Deeming *et al.* (1978 ▶); Bose & Saha (1987 ▶). For crystal structures of neutral pyridine-palladacycles, see: Lu *et al.* (2005 ▶); Fun *et al.* (2006 ▶). For an approach to the study of the relative binding affinities of unidentate ligands for organopalladium(II) species, see: Hoffman *et al.* (2009 ▶).
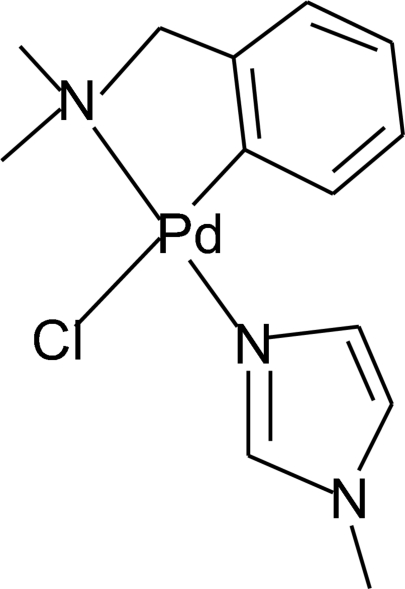


## Experimental

### 

#### Crystal data

[Pd(C_9_H_12_N)Cl(C_4_H_6_N_2_)]
*M* *_r_* = 358.15
Orthorhombic, 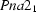

*a* = 25.5485 (15) Å
*b* = 10.0057 (6) Å
*c* = 5.6733 (4) Å
*V* = 1450.27 (16) Å^3^
*Z* = 4
Mo *K*α radiation
μ = 1.45 mm^−1^
*T* = 290 K
0.43 × 0.15 × 0.09 mm

#### Data collection

Oxford Diffraction Xcalibur E diffractometer
Absorption correction: multi-scan (*CrysAlis PRO*; Oxford Diffraction, 2010 ▶) *T*_min_ = 0.788, *T*_max_ = 1.00
6592 measured reflections
2373 independent reflections
2057 reflections with *I* > 2σ(*I*)
*R*_int_ = 0.028

#### Refinement

*R*[*F* ^2^ > 2σ(*F* ^2^)] = 0.022
*wR*(*F* ^2^) = 0.044
*S* = 0.96
2373 reflections
167 parameters
1 restraint
H-atom parameters constrained
Δρ_max_ = 0.31 e Å^−3^
Δρ_min_ = −0.38 e Å^−3^
Absolute structure: Flack (1983 ▶), 852 Friedel pairs
Flack parameter: −0.04 (3)

### 

Data collection: *CrysAlis PRO* (Oxford Diffraction, 2010 ▶); cell refinement: *CrysAlis PRO*; data reduction: *CrysAlis PRO*; program(s) used to solve structure: *SHELXS97* (Sheldrick, 2008 ▶); program(s) used to refine structure: *SHELXL97* (Sheldrick, 2008 ▶); molecular graphics: *OLEX2* (Dolomanov *et al.*, 2009 ▶); software used to prepare material for publication: *publCIF* (Westrip, 2010 ▶).

## Supplementary Material

Crystal structure: contains datablocks I, global. DOI: 10.1107/S1600536810047367/ng5067sup1.cif

Structure factors: contains datablocks I. DOI: 10.1107/S1600536810047367/ng5067Isup2.hkl

Additional supplementary materials:  crystallographic information; 3D view; checkCIF report

## Figures and Tables

**Fig. 1.**
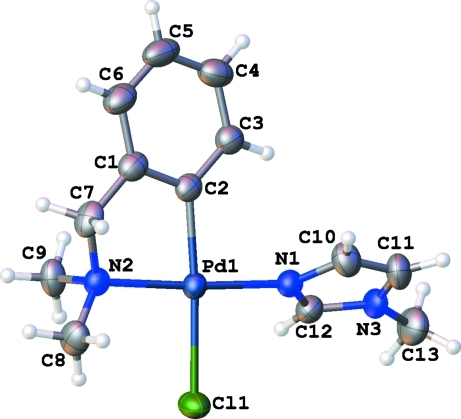
A thermal ellipsoid plot (50%) of I showing the labeling scheme.

## supplementary crystallographic information

### Comment

Palladacycles are an important class of catalysts for organic reactions (DuPont & Flores, 2009; Bedford *et al.*, 2003; Fors & Buchwald, 2010), including methanolysis of phosphorothionate pesticides (Lu *et al.*, 2005; Lu *et al.*, 2010). One of the most commonly used, and now commercially available, palladacyclic dimers, di-*µ*-chlorobis[2-(dimethylamino)benzyl-*κ^2^C^1^*,*N*]palladium(II), or [(*κ^2^*-dmba)PdCl]_2_ for short, was first prepared by Cope & Friedrich (1968), with its structure solved by Mentes, Kemmitt, *et al.* (2004). Many compounds of the general formula (*κ^2^*-dmba)Pd(*L*)Cl are easily prepared by treating this dimer with two molar equivalents of neutral unidentate ligand, *L*. The great majority of these products contain pnictogen ligands, primarily phosphines; crystal structures have been published for *L* = PPh_3_ (Mentes, Kemmitt, *et al.*, 2004) and SbPh_3_ (Mentes & Büyükgüngör, 2004). Combining four molar equivalents of these triphenylpnictogens with the dimer affords dechelation of the dmba moiety and formation of the square-planar *trans*-(EPh_3_)_2_Pd(2-dmba-*κC^1^*)Cl. Relatively few examples of (*κ^2^*-dmba)Pd(N-ligand)Cl have been reported, and those are almost exclusively in the pyridine family (Deeming *et al.*, 1978; Bose & Saha, 1987), with crystal structures reported for the pyridine (Lu *et al.*, 2005) and 4-dimethylaminopyridine (Fun *et al.*, 2006) complexes.

Our interest in studying relative binding affinities of soft metal centers for ligands of moderate and weak donor power using ^19^F and ^31^P NMR spectroscopy (Hoffman *et al.*, 2009) to monitor ligand-substitution equilibria led us to prepare the title complex (I), whose structure is shown in Figure 1. Suitable single crystals were grown from vapor diffusion of heptane into a solution of the 1-methylimidazole complex at room temperature. All four Pd-ligand bond lengths were similar to those reported for other (*κ^2^*-dmba)Pd(*L*)Cl structures, especially those for the two pyridine-family complexes (Lu *et al.*, 2005; Fun *et al.*, 2006). The angle between the imidazole ring and the palladacycle plane (Pd1–N2–C1–C2–C7) in I is 57.88 (16)°, on par with the 49.2° angle between the pyridine and palladacycle rings in (*κ^2^*-dmba)Pd(py)Cl (Lu *et al.*, 2005). However, both these angles are quite smaller than the comparable dihedral angles in (*κ^2^*-dmba)Pd(dmap)Cl (dmap = 4-(dimethylamino)pyridine) (Fun *et al.*, 2006) for which three crystallographically independent molecules yielded values of 76.80 (14)°, 81.85 (14)°, and 83.74 (14)°.

### Experimental

To a solution of 0.100 mmol [(*κ^2^*-C_9_H_12_N)PdCl]_2_ (Sigma-Aldrich) in 2.0 ml e thanol-free reagent chloroform (Fisher) in a 10-ml glass vial was added with stirring 0.200 mmol neat 1-methylimidazole (Sigma-Aldrich). The resulting pale-yellow solution was subjected to vapor diffusion with 30 ml heptane (Fisher reagent) at room temperature for 3 days. The small amount of liquid remaining was removed by disposable glass pipet from the resulting off-white needles, and the crystals were washed twice with 5.0 ml of hexanes (Fisher reagent). All reagents and solvents were used as received. The desired needles were removed from the vial and air-dried overnight in the dark (94% yield).

### Refinement

Hydrogen atoms were placed in calculated positions and allowed to ride during subsequent refinement, with *U*_iso_(H) = 1.2*U*_eq_(C) and C—H distances of 0.93 Å for the ring H atoms, *U*_iso_(H) = 1.2*U*_eq_(C) and C—H distances of 0.97 Å for the methylene H atoms, and *U*_iso_(H) = 1.5*U*_eq_(C) and C—H distances of 0.96 Å for the methyl H atoms.

### Crystal data

**Table:**

[Pd(C_9_H_12_N)Cl(C_4_H_6_N_2_)]	*F*(000) = 720
*M**_r_* = 358.15	*D*_x_ = 1.640 Mg m^−^^3^
Orthorhombic, *P**n**a*2_1_	Mo *K*α radiation, λ = 0.71073 Å
Hall symbol: P 2c -2n	Cell parameters from 3299 reflections
*a* = 25.5485 (15) Å	θ = 3.1–25.6°
*b* = 10.0057 (6) Å	µ = 1.45 mm^−^^1^
*c* = 5.6733 (4) Å	*T* = 290 K
*V* = 1450.27 (16) Å^3^	Prism, colorless
*Z* = 4	0.43 × 0.15 × 0.09 mm

### Data collection

**Table:**

Oxford Diffraction Xcalibur E diffractometer	2373 independent reflections
Radiation source: fine-focus sealed tube	2057 reflections with *I* > 2σ(*I*)
graphite	*R*_int_ = 0.028
Detector resolution: 16.0514 pixels mm^-1^	θ_max_ = 25.7°, θ_min_ = 3.1°
ω scans	*h* = −31→31
Absorption correction: multi-scan (*CrysAlis PRO*; Oxford Diffraction, 2010)	*k* = −12→10
*T*_min_ = 0.788, *T*_max_ = 1.00	*l* = −6→6
6592 measured reflections	

### Refinement

**Table:**

Refinement on *F*^2^	Hydrogen site location: inferred from neighbouring sites
Least-squares matrix: full	H-atom parameters constrained
*R*[*F*^2^ > 2σ(*F*^2^)] = 0.022	*w* = 1/[σ^2^(*F*_o_^2^) + (0.0205*P*)^2^] where *P* = (*F*_o_^2^ + 2*F*_c_^2^)/3
*wR*(*F*^2^) = 0.044	(Δ/σ)_max_ = 0.003
*S* = 0.96	Δρ_max_ = 0.31 e Å^−^^3^
2373 reflections	Δρ_min_ = −0.38 e Å^−^^3^
167 parameters	Extinction correction: *SHELXL97* (Sheldrick, 2008), Fc^*^=kFc[1+0.001xFc^2^λ^3^/sin(2θ)]^-1/4^
1 restraint	Extinction coefficient: 0.0038 (2)
Primary atom site location: structure-invariant direct methods	Absolute structure: Flack (1983), 852 Friedel pairs
Secondary atom site location: difference Fourier map	Flack parameter: −0.04 (3)

### Special details

**Table:**

Geometry. All e.s.d.'s (except the e.s.d. in the dihedral angle between two l.s. planes) are estimated using the full covariance matrix. The cell e.s.d.'s are taken into account individually in the estimation of e.s.d.'s in distances, angles and torsion angles; correlations between e.s.d.'s in cell parameters are only used when they are defined by crystal symmetry. An approximate (isotropic) treatment of cell e.s.d.'s is used for estimating e.s.d.'s involving l.s. planes.
Refinement. Refinement of *F*^2^ against ALL reflections. The weighted *R*-factor *wR* and goodness of fit *S* are based on *F*^2^, conventional *R*-factors *R* are based on *F*, with *F* set to zero for negative *F*^2^. The threshold expression of *F*^2^ > 2σ(*F*^2^) is used only for calculating *R*-factors(gt) *etc*. and is not relevant to the choice of reflections for refinement. *R*-factors based on *F*^2^ are statistically about twice as large as those based on *F*, and *R*- factors based on ALL data will be even larger.

### Fractional atomic coordinates and isotropic or equivalent isotropic displacement parameters (Å^2^)

**Table:**

	*x*	*y*	*z*	*U*_iso_*/*U*_eq_	
Pd1	0.644535 (8)	0.91814 (2)	0.75150 (8)	0.02903 (8)	
Cl1	0.67437 (4)	0.69431 (8)	0.66642 (19)	0.0466 (3)	
C1	0.65047 (13)	1.1994 (4)	0.6894 (7)	0.0385 (12)	
C2	0.62563 (13)	1.1054 (3)	0.8301 (6)	0.0331 (9)	
C3	0.59495 (14)	1.1503 (4)	1.0170 (7)	0.0378 (9)	
H3	0.5781	1.0885	1.1131	0.045*	
C4	0.58921 (15)	1.2846 (4)	1.0615 (8)	0.0494 (11)	
H4	0.5694	1.3131	1.1894	0.059*	
C5	0.61300 (17)	1.3768 (4)	0.9156 (9)	0.0560 (13)	
H5	0.6084	1.4677	0.9432	0.067*	
C6	0.64348 (13)	1.3354 (3)	0.7301 (14)	0.0507 (11)	
H6	0.6594	1.3979	0.6322	0.061*	
C7	0.68436 (15)	1.1482 (4)	0.4924 (7)	0.0424 (10)	
H7A	0.7140	1.2075	0.4690	0.051*	
H7B	0.6644	1.1446	0.3470	0.051*	
C8	0.71890 (16)	0.9419 (4)	0.3383 (7)	0.0460 (10)	
H8A	0.7460	0.9922	0.2615	0.069*	
H8B	0.6892	0.9346	0.2354	0.069*	
H8C	0.7317	0.8541	0.3754	0.069*	
C9	0.74967 (12)	1.0235 (3)	0.7096 (7)	0.0422 (11)	
H9A	0.7773	1.0667	0.6236	0.063*	
H9B	0.7610	0.9364	0.7585	0.063*	
H9C	0.7410	1.0760	0.8458	0.063*	
C10	0.53338 (14)	0.8549 (4)	0.9384 (8)	0.0464 (10)	
H10	0.5162	0.9102	0.8317	0.056*	
C11	0.51014 (15)	0.7778 (4)	1.1018 (7)	0.0475 (11)	
H11	0.4744	0.7707	1.1295	0.057*	
C12	0.59415 (13)	0.7524 (3)	1.1267 (7)	0.0397 (9)	
H12	0.6268	0.7233	1.1774	0.048*	
C13	0.54318 (17)	0.6178 (4)	1.4131 (8)	0.0577 (12)	
H13A	0.5750	0.5678	1.4306	0.087*	
H13B	0.5148	0.5577	1.3805	0.087*	
H13C	0.5361	0.6658	1.5561	0.087*	
N1	0.58669 (10)	0.8386 (3)	0.9548 (6)	0.0344 (7)	
N2	0.70326 (11)	1.0106 (3)	0.5575 (5)	0.0312 (7)	
N3	0.54872 (11)	0.7121 (3)	1.2190 (7)	0.0388 (8)	

### Atomic displacement parameters (Å^2^)

**Table:**

	*U*^11^	*U*^22^	*U*^33^	*U*^12^	*U*^13^	*U*^23^
Pd1	0.02726 (11)	0.02814 (13)	0.03167 (13)	−0.00098 (10)	0.0002 (2)	0.0043 (2)
Cl1	0.0495 (5)	0.0321 (5)	0.0582 (7)	0.0077 (4)	0.0119 (5)	0.0049 (4)
C1	0.0352 (19)	0.034 (2)	0.047 (4)	0.0014 (15)	−0.0090 (17)	0.0041 (17)
C2	0.0269 (16)	0.032 (2)	0.041 (3)	0.0007 (15)	−0.0080 (15)	0.0031 (16)
C3	0.034 (2)	0.037 (2)	0.043 (3)	0.0004 (17)	−0.0025 (18)	0.0003 (19)
C4	0.046 (2)	0.044 (3)	0.058 (3)	0.009 (2)	−0.004 (2)	−0.011 (2)
C5	0.048 (2)	0.033 (2)	0.087 (4)	0.0030 (19)	−0.018 (3)	−0.009 (2)
C6	0.0475 (19)	0.033 (2)	0.071 (3)	0.0001 (16)	−0.008 (3)	0.006 (3)
C7	0.044 (2)	0.042 (2)	0.041 (3)	−0.0068 (18)	−0.006 (2)	0.020 (2)
C8	0.051 (2)	0.053 (2)	0.034 (2)	−0.0084 (18)	0.0101 (18)	0.0017 (18)
C9	0.0295 (16)	0.060 (2)	0.037 (3)	−0.0068 (15)	−0.002 (2)	0.006 (2)
C10	0.034 (2)	0.053 (3)	0.052 (3)	0.0031 (18)	0.000 (2)	0.006 (2)
C11	0.031 (2)	0.056 (3)	0.055 (3)	−0.0065 (19)	0.006 (2)	0.002 (2)
C12	0.036 (2)	0.032 (2)	0.051 (2)	−0.0019 (17)	0.0034 (18)	0.000 (2)
C13	0.062 (3)	0.054 (3)	0.057 (3)	−0.007 (2)	0.018 (2)	0.013 (2)
N1	0.0316 (16)	0.0307 (17)	0.0410 (19)	−0.0003 (13)	0.0020 (14)	0.0018 (15)
N2	0.0309 (15)	0.0324 (17)	0.0302 (18)	−0.0020 (13)	−0.0020 (13)	0.0029 (14)
N3	0.0405 (15)	0.0363 (15)	0.039 (2)	−0.0060 (11)	0.0153 (18)	0.0031 (18)

### Geometric parameters (Å, °)

**Table:**

Pd1—C2	1.985 (3)	C8—H8A	0.9600
Pd1—N1	2.037 (3)	C8—H8B	0.9600
Pd1—N2	2.078 (3)	C8—H8C	0.9600
Pd1—Cl1	2.4145 (9)	C9—N2	1.472 (4)
C1—C2	1.388 (5)	C9—H9A	0.9600
C1—C6	1.391 (5)	C9—H9B	0.9600
C1—C7	1.504 (5)	C9—H9C	0.9600
C2—C3	1.393 (5)	C10—C11	1.345 (5)
C3—C4	1.375 (5)	C10—N1	1.375 (4)
C3—H3	0.9300	C10—H10	0.9300
C4—C5	1.381 (6)	C11—N3	1.358 (5)
C4—H4	0.9300	C11—H11	0.9300
C5—C6	1.373 (8)	C12—N1	1.316 (4)
C5—H5	0.9300	C12—N3	1.335 (4)
C6—H6	0.9300	C12—H12	0.9300
C7—N2	1.505 (4)	C13—N3	1.457 (5)
C7—H7A	0.9700	C13—H13A	0.9600
C7—H7B	0.9700	C13—H13B	0.9600
C8—N2	1.476 (4)	C13—H13C	0.9600
			
C2—Pd1—N1	93.72 (13)	H8B—C8—H8C	109.5
C2—Pd1—N2	82.79 (13)	N2—C9—H9A	109.5
N1—Pd1—N2	176.23 (11)	N2—C9—H9B	109.5
C2—Pd1—Cl1	175.52 (10)	H9A—C9—H9B	109.5
N1—Pd1—Cl1	88.84 (8)	N2—C9—H9C	109.5
N2—Pd1—Cl1	94.54 (8)	H9A—C9—H9C	109.5
C2—C1—C6	120.6 (4)	H9B—C9—H9C	109.5
C2—C1—C7	117.4 (3)	C11—C10—N1	108.8 (4)
C6—C1—C7	122.0 (4)	C11—C10—H10	125.6
C1—C2—C3	118.4 (3)	N1—C10—H10	125.6
C1—C2—Pd1	113.5 (3)	C10—C11—N3	107.1 (3)
C3—C2—Pd1	127.8 (3)	C10—C11—H11	126.4
C4—C3—C2	121.0 (4)	N3—C11—H11	126.4
C4—C3—H3	119.5	N1—C12—N3	111.2 (3)
C2—C3—H3	119.5	N1—C12—H12	124.4
C3—C4—C5	119.7 (4)	N3—C12—H12	124.4
C3—C4—H4	120.1	N3—C13—H13A	109.5
C5—C4—H4	120.1	N3—C13—H13B	109.5
C6—C5—C4	120.5 (4)	H13A—C13—H13B	109.5
C6—C5—H5	119.8	N3—C13—H13C	109.5
C4—C5—H5	119.8	H13A—C13—H13C	109.5
C5—C6—C1	119.7 (5)	H13B—C13—H13C	109.5
C5—C6—H6	120.1	C12—N1—C10	105.8 (3)
C1—C6—H6	120.1	C12—N1—Pd1	124.8 (2)
C1—C7—N2	108.3 (3)	C10—N1—Pd1	129.3 (3)
C1—C7—H7A	110.0	C9—N2—C8	108.5 (3)
N2—C7—H7A	110.0	C9—N2—C7	108.8 (3)
C1—C7—H7B	110.0	C8—N2—C7	107.8 (3)
N2—C7—H7B	110.0	C9—N2—Pd1	108.1 (2)
H7A—C7—H7B	108.4	C8—N2—Pd1	115.7 (2)
N2—C8—H8A	109.5	C7—N2—Pd1	107.8 (2)
N2—C8—H8B	109.5	C12—N3—C11	107.0 (3)
H8A—C8—H8B	109.5	C12—N3—C13	125.2 (3)
N2—C8—H8C	109.5	C11—N3—C13	127.8 (3)
H8A—C8—H8C	109.5		
